# Energy excess is the main cause of accelerated aging of mammals

**DOI:** 10.18632/oncotarget.4271

**Published:** 2015-05-26

**Authors:** Tomasz Biliński, Tadeusz Paszkiewicz, Renata Zadrag-Tecza

**Affiliations:** ^1^ Department of Biochemistry and Cell Biology, University of Rzeszow, Rzeszow, Poland; ^2^ Retired from Rzeszów University of Technology, Rzeszow, Poland

**Keywords:** aging, longevity, energy, glyco/lipotoxicity, epigenetic maintenance system

## Abstract

The analysis of cases of unusually high longevity of naked mole rats and an alternative explanation of the phenomenon of calorie restriction effects in monkeys allowed for postulating that any factor preventing an excess of energy consumed, leads to increased lifespan, both in evolutionary and an individual lifetime scale. It is postulated that in mammals the most destructive processes resulting in shortening of life are not restricted to the phenomena explained by the hyperfunction theory of Mikhail Blagosklonny. Hyperfunction, understood as unnecessary or even adverse syntheses of cell components, can be to some extent prevented by lowered intake of nutrients when body growth ceases. We postulate also the contribution of glyco/lipotoxicity to aging, resulting from the excess of energy. Besides two other factors seem to participate in aging. One of them is lack of telomerase activity in some somatic cells. The second factor concerns epigenetic phenomena. Excessive activity of epigenetic maintenance system probably turns off some crucial organismal functions. Another epigenetic factor playing important role could be the micro RNA system deciding on expression of numerous age-related diseases. However, low extrinsic mortality from predation is a *conditio sine qua non* of the expression of all longevity phenotypes in animals. Among all long-lived animals, naked mole rats are unique in the elimination of neoplasia, which is accompanied by delayed functional symptoms of senescence. The question whether simultaneous disappearance of neoplasia and delayed senescence is accidental or not remains open.

## INTRODUCTION

To date, over 300 theories explaining aging were put forward. Some of them, like the uncritically accepted free radical theory of aging [1], do not find unequivocal experimental support [2]. Others, like Weissman's distinction between mortal soma and immortal germ line or disposable soma theory [3], can explain only general rules of aging, but are restricted to animals. Those, like antagonistic pleiotropy theory [4], are informative, but cannot explain the details of mechanisms of senescence and longevity. The closest to ideas presented in this paper is the postulate of hyperfunction [2, 5–7].

Another factor slowing down the development of gerontological studies is a semantic chaos which resulted in confusion similar to biblical Tower of Babel effect. Leonard Hayflick mentioned that “the dozen or more experts do not agree on the definition of basic gerontological terms, including aging”. His sarcastic opinions are best summarized by the title of his article: “Entropy Explains Aging, Genetic Determinism Explains Longevity, and Undefined Terminology Explains Misunderstanding Both” [8].

Under these circumstances we decided to rarely use the term “aging” as too general, and throughout this paper we will use mostly terms “senescence” and “longevity”, both fitting the term “aging”. Senescence refers to all mechanisms and consequences of wear and tear processes, whereas longevity of the populations should be established exclusively by the length of life of individuals, whose death was unambiguously confirmed. Hence, for example ceasing reproduction by yeast cell does not mean its death [9–11]. Short glossary provided at the end of this article should make further considerations more clear.

The most important factor negatively influencing gerontological studies was a belief best expressed in two sentences: “Finally, we believe there is good evidence for universal mechanisms of aging (at least between fungi and metazoans). Parsimony suggests that the ubiquity of aging is likely the result of conserved mechanisms of aging” [12]. This type of reasoning is fundamentally erroneous; as similar reasoning error, the ubiquity of sounds in our neighborhood is not the proof of their common origin or mechanism. However, the basic fallacy of this assumptions derives from that aging is ubiquitous mainly among animals. Even in birds the phenomenon is already not so obvious. In numerous species of taxonomic groups of animals, beginning with frequently “immortal” cnidarians through echinoderms, mollusks, crustaceans fishes and amphibians until, reptiles, the symptoms of senescence are hard to find or simply do not exist. This clearly *ad hoc*, erroneous assumption was used to validate the use of evolutionary and structurally distant species, like unicellular yeast as a model organism of gerontology to explain aging of complex organisms, including humans. It happened irrespective of numerous substantial reservations [9, 13, 14].

This opinion, however, contrasts in an obvious way with the practice of comparing closely related organisms of the similar size, which is widely accepted among animal gerontologists. We have recently presented numerous arguments that both senescence and the mechanism leading to death of organisms evolved in parallel to the evolution of diverse life forms [15].

In recent analysis, we decided to focus primarily on unquestionable experimental facts, while simultaneously trying to eliminate all unnecessary accretions that accumulated during many years of studies. The hypothesis that we put forward is based on the analysis of unusual cases of extraordinary longevity of selected mammals. Besides, we took into account the procedures which are known to extend longevity of various organisms including human beings. First of them is Mediterranean diet [16, 17] and the second one is known as calorie restriction regime [18, 19].

## CASES OF UNUSUALLY HIGH LONGEVITY

Examples of unusually long-living organisms can be found both in typical and atypical environments. High longevity of organisms living in atypical environments is well known. It concerns selective cases, like deep see and saline or freshwater cave species. Terrestrial examples are represented by organisms living in a deep burrow system, like the naked mole rats (NMRs) living in the eastern corner of Africa. These animals, in contrast to other rodents living in burrows, seal them, creating their own unique ecosystem. These animals live nearly thirty years [20], whereas most of similar rodents of the same size live at the most several years, and in practice much shorter. Much more frequent are long-lived organisms occupying more typical environments of the Earth. The examples of unusually long life are most frequent in some arthropod groups, like crustaceans, and also among lower vertebrates. In this paper we will, however, concentrate exclusively on mammals and human beings, although some of the general conclusions drawn are applicable to most animals. The only exceptions are the simplest animals, like some cnidarians that are biologically immortal [21].

## CALORIE RESTRICTION AS THE ONLY UNIVERSAL LIFESPAN EXTENDING REGIME

Human beings inhabit almost all continents and a variety of climates. This allows one to draw the conclusion that some nutritional habits can measurably increase individual lifespan, irrespective of differences in origin.

Mediterranean diet is known to extend the lifespan of human beings [16, 17]. However, the more famous of the two feeding regimes that have been statistically proven to extend lifespan in a wide range of organisms is calorie restriction [18, 22, 23]. This regime consists in ca. 30% decrease of calorie intake, when other nutrients are available in satisfactory amounts [24]. However, the negative effects of calorie restriction on the quality of life, including reduced libido, are hardly acceptable to an average person, and thus it is not likely to be implemented [25, 26]. Further, the conclusion that calorie restriction really extends the lifespan of our cousins *Macaca mulatta* has recently become less obvious [27].

Below, we propose an alternative explanation of recent [24, 27] experimental data, which is one of the elements of our hypothesis. The controversies result from not fully rational establishing of the control sample at the beginning of the studies. Lifespan extending effects are observed only if the control for calorie restriction consists in feeding animals *ad libitum*. In addition, the tasty fodder was used. The differences were not observed if the control animal population was fed according to the procedure adapted to the need of organisms. Therefore, instead postulating that calorie restriction extends lifespan, one can even more logically postulate that feeding *at libitum* results in adverse effects. Consequently, it is not that calorie restriction prolongs life, but that the excess of calories shortens it. This alternative explanation of calorie restriction effects suggested to us the need for analysis of the possibility that unusually high longevity of NMRs, when compared to similar rodents, also can result from preventing the energy surplus. Unusually high, in this context, means longevity much higher than that of other closely related species of similar size and showing similar life strategies. Comparison of evolutionary distant organisms is to a high extent risky.

It wasn't only the effects of calorie restriction on monkeys that suggested such explanation. It was earlier proposed [28] that if an individual has an excess of energy, its flow is not optimal. This Author concluded that the general quality of living system is destabilized by an excess energy production, which influenced the rate of senescence and longevity. The Author, however, ascribed the negative consequences to the increased rate of free radical production [28], which hardly finds support in the results of recent experiments [29].

## GROWTH OF LIVING THINGS IS ACCOMPANIATED BY INTERNAL ENTROPY CHANGE AND DISSIPATION

The cited title of L. Hayflick article [8] suggests a principal role of entropy in the aging process, which contrasts with a rather limited role of actual damage or accumulating damage in aging [30]. Therefore, explaining the role of entropy in living systems seems to be worthwhile [31]. Living systems are open systems, which invariably operate very far from thermodynamic equilibrium. They are in contact with an environment (a bath), exchanging with it energy and matter.

Styer [32] and Bunn [33] made quantitative estimations of the entropy involved in the biological evolution and demonstrated that there is no conflict between evolution and the second law of thermodynamics. Their estimations clearly indicate that the emergence of living species and their evolution involves flow of negative entropy. Although entropy must increase over time in an isolated (closed) system, an open system can keep its entropy low - that is, divide energy unevenly among its elements - by increasing the entropy of its surroundings.

In Karolinska and Technion talks, England (England talks) states that “living things tend towards ‘low’ internal entropy, are ‘durable’ on growth timescale, get the environment to do work on them, and then dissipate it back in the form of heat. Thus, the growth of living things is accompanied by internal entropy change and dissipation”. He proposed a generalization of the second law of thermodynamics that holds for systems which are strongly driven by an external energy source, such as an electromagnetic radiation, and they can in an irreversible way dispose heat into a surrounding bath. Let us present his formulation of the second law of thermodynamics.

Assume that π(I→II) is the probability that a macroscopic system initially prepared in a state I propagated over a certain period of time and was then found to be in a state II. The second probability π(II→I) gives the likelihood that after another interval of, the same system would be observed again in the state I.

England's [34] formulation of the second law of thermodynamics accounts for contribution to entropy proportional to [π(II→I)/π(I→II)], the change of entropy of the bath $\Delta Q_{1\to II}/T$, and the change in the entropy of the system $\Delta S_{i\text{nt}}=\left(S_{II}-S_{I}\right)$. T is the temperature of the bath. If π(II→I)<π(I→II), the logarithmic term is negative. Therefore, we shall call the suitable contribution to entropy the negentropy. Bunn relates it with life and denotes $\Delta S_{life}$ [33].

The more likely an initial state I is than the state II, the more negative is the negentropy. According the second law of thermodynamics, the sum of these three terms have to be nonnegative

$$\frac{\Delta Q_{I\to II}}{T}+k_{B}\ln\left[\frac{\pi \left(II\to I\right)}{\pi \left(I\to II\right)}\right]+\Delta S_{\mathrm{int}}\geq 0,$$

where $K_{B}=1.38\times 10^{-23}\;J/K$ is the Boltzmann constant and T. $\Delta S_{\mathrm{int}}$ is the entropy generated in physiological processes accompanying evolution processes towards the state II. Evolution from less complicated state I to more complicated state II is possible, if there is a *flow of energy* and a *program of development.*

In the absence of the second term, i.e. when π(I→II)=π(II→I), England's inequality reduces to the standard formulation of the second law of thermodynamics: the change of the entropy of the system $\Delta S_{\mathrm{int}}$ plus the change in the entropy of the bath $\Delta Q_{I\to II}/T$ must be greater or equal to zero.

From England's inequality, we conclude that the more irreversible a process is (i.e. more negative negentropic term is), the more positive must be the minimum total entropy production $\left(\Delta Q_{I\to II}/T+\Delta S_{\mathrm{int}}\right)$.

Hayflick [8] attributes the entropy production only to molecular processes. However, these processes contribute to $\Delta S_{\mathrm{int}}$. England's approach allows one to consider the processes on the macroscopic level. For example, he was able to consider the bacterial cell division.

## TWO ENVIRONMENTAL MECHANISMS ACTING TOGETHER ENABLE LONGEVITY OF NMRs

Gerontological theories rarely take into account the simple fact that all living things that presently exist have a long evolutionary history which has influenced the length of their lifespan. Therefore, the analysis of environmental factors which influenced the actual lifespan of a population would help in understanding recent biology of particular species. The analysis of life of naked mole rats (NMRs) is the best illustration of how strongly environmental factors can influence evolution of longevity. To much lower extent, they influence longevity of actual populations.

NMRs belong to rodents, which with a few exceptions of their bigger representatives, like capybara, have a very short lifespan. Ecologists suggest that longevity as well as fecundity of animals depend on extrinsic mortality from predation [35]. In contrast to NMRs, other rodents living also in soil leave their burrows very frequently for food, which is much more abundant on the surface where numerous predators hunt. Others, living in shallow burrows, profit from much higher abundance of food just under the soil surface, in contrast to deeper burrow dwellers like NMRs. Rodents like voles are also an easy prey of numerous predators, because shallow burrows are easily penetrated by small mammalian predators or snakes. Consequently, life in deep, sealed burrow systems assures much higher protection against predators, which is strongly enforced by eusociality of some NMR species [36]. Workers/soldiers additionally protect the most endangered places of the burrow system. The role of low predation pressure is a prerequisite of increased longevity. Similar, but much less high life extension effects were found in opossum living on predator-free islands [37].

However, the choice of life in deep sealed burrows as the only habitat, while bringing about higher protection against pathogens, also results in one important limitation. These animals feed mainly on deeply located, large tubers. The rate of this food renewal is therefore extremely low. This is the price paid for increased safety. The consequences are as follows: growth of young animals, as well as their maturation, requires much more time because of scarcity of food. Their metabolic rate is lowered. This scarcity causes the frequency of litters to be reduced to one per year, instead of several of them in other rodents. Thus, NMRs cannot only rely on increasing the number of progeny in a single litter as a viable reproductive strategy due to of the lack of resources; consequently their strategy consists in prolonging their period of sexual reproduction for a number of years to assure the appropriate number of progeny. This need required the elimination of inherited mutations or gene variants increasing the rate of aging, including senescence of reproductory system. Under the pressure of the NMR's habitat, only the animals with fewer life shortening genes could effectively reproduce. The longer an individual's lifespan, the more prevalent its genome has become. Therefore, the two factors: low extrinsic mortality from predation and low rate of food renewal caused NMRs to live up to ca. 30 years instead of two or three years characteristic for other rodents. The longevity of NMRs is accompanied by the late onset of functional symptoms of senescence and complete elimination of neoplasia accompanied by a decrease in other typical age-related diseases [38, 39].

The mechanism of low food availability accompanied by the elimination or substantial lowering of extrinsic mortality from predation is also useful in explaining other known cases of extraordinary longevity of animals living in other terrestrial environments, like wood logs and soil. Life in three earlier mentioned aquatic environments depends also on rather unpredictable and constant low external sources of food.

## POSSIBLE MECHANISMS OF PREVENTING ENERGY SURPLUS IN ANIMALS LIVING IN TYPICAL ENVIRONMENTS

### Body size growth as an energy security valve is one of possible mechanisms enabling longevity

Examples of unusually high longevity of animals occupying ecosystems which are not so strongly energy limited are much more numerous. The best known is longevity of some arthropods, big fishes, amphibians and giant turtles. The fish *Hoplostethus atlanticus* is probably one the longest recently vertebrates (150 years or more) [40, 41] among vertebrates. Bivalve edible mollusk *Arctica islandica* lives for more than 500 years [42]. Finding a common denominator for these diverse organisms is not easy. However, if we analyze their lifestyles, two types of life strategies become clear. Almost all of them, except mammals and birds, increase their size during the whole life, i.e. even after reaching maturity. One can postulate that negative consequences of energy surplus can be prevented by spending extra energy on very costly constant body growth. Therefore, the strategy of continuous body size increase can be treated as a “security valve” preventing energy excess. This strategy, so different from that of mammals, will be discussed in the our forthcoming paper.

### Evolution of energy dissipation mechanism can extend lifespan, likewise playing the role of security valve

However, of most interest to human beings, “warm blooded” (homeothermic) mammals, is our own fate. We stop growing soon after reaching sexual maturity. The energy needs of adult mammal individuals decrease significantly when compared to the period of adolescence, except during periods of pregnancy and lactation. The molecular consequences of this fact were analyzed by M.V. Blagosklonny [2, 6, 30, 43, 44]. These organisms lost this energy security valve when evolving from reptiles. Birds and mammals, however, arrived at the mechanism of temperature control set at the level balancing the benefits resulting from the increased rate of biological processes and the risks of denaturation of bio molecules by heat resulting from the increase in temperature. Consequently, an alternative system of energy excess expenditure by energy dissipation appeared as a side effect, giving homeothermic organisms a strong advantage over other animals. Hence, longevity of mammals and birds can be treated as a side effect of evolving the mechanisms of maintaining constantly high temperature [45, 46]. How important for energy expenditure was the development of temperature regulation mechanisms is best visualized by the following comparison based on various data. To produce one kilogram of carp, we need up to 4 times more fodder than to produce the same weight of waterfowl feeding in similar environment. However, under optimal thermal conditions this difference drops down substantially. Energetic costs of synthesis of biomass of both species are to a high extent comparable, hence the difference results from thermoregulation and its consequences.

As a consequence, we consider mainly increased consumption of energy, spent for incomparably higher motility and functioning of enormously efficient muscles. Increased motility or other types of energy dissipation through the action of muscles and functioning of brain, further triggers longevity mechanisms, independent of other strategies. High investment in various types of physical exercise is one of additional factors promoting longevity. It is visible in many groups of long-lived organisms. For example, life underground requires high energy investment in digging burrows to find food. The same is true in case of insect larvae living in wood. Life in Sahel requires enormous investment in constant migration, etc. Lightweight plumage is important for flight and insulation, but is strongly vulnerable to molds, and numerous parasites especially in wet environments. Molting and care of plumage takes most of the resting time of birds like parrots and uses additional resources. Comparatively high longevity of birds can result also from additional energy expenditure resulting from the body temperature higher than in mammals.

Physical activity of homeotherms is incomparable to activity of cold-blooded (poikilothermic) animals. Therefore, lack of security valve in the form of investment in constant growth is recompensed in mammals and birds by increased energy expenditure in the form of new, very profitable mechanisms of energy dissipation.

Increased longevity of females is observed not only in case of human beings, but also in other quite evolutionarily distant species [47–49]. The differences between sexes are usually not as spectacular as the differences between species, but statistically meaningful. This observation can be at least partially explained by the energy surplus hypothesis. It is obvious that an investment in eggs, which are large and rich in nutrients, requires much more energy expenditure than the formation of sperm. In mammals, an additional investment in progeny is necessary during pregnancy and lactation. Consequently, the metabolism of females should be better tuned to adapt to changing needs than the male metabolism. The phenomenon of female longevity is observed also in various groups of animals.

## POSSIBLE BIOCHEMICAL OR PHYSIOLOGICAL MECHANISMS SHORTENING LIFESPAN

Mikhail Blagosklonny [5, 6] postulates that when adolescence ends, an organism instead ceasing various biosynthetic processes continue them, which leads to cell hypertrophy with its all negative consequences. According to Gems and de la Guardia [2], he introduced the term bloated soma, which visualizes the problem. Therefore, the idea of energy excess is in line with his revolutionary thoughts.

The postulate of the strong impact of energy excess on longevity of mammals or birds opens the discussion on its molecular mechanism. The only other known and generally accepted factor potentially increasing longevity of human beings is the Mediterranean diet [17]. To counterbalance the opinion that energy excess acts through the action of free radicals as proposed by Melamede [28], we propose much more trivial physiological or biochemical mechanisms. We propose to interpret the effects of energy excess according the following series of arguments: Wild animals encounter periods of plenty and food scarcity. Therefore, to survive periods of sometimes very strong starvation, the mechanisms of mainly fat storage evolved, on both cellular and organismal levels. These mechanisms assure survival during periods of scarcity. Awareness of the negative consequences of prolonged or constantly high food availability has recently become widespread in developed countries, but the understanding of the essence of the problem is not equally common. An obvious and easily understandable consequence of excess of food is overweight. Obese people become more obese, because overweight decreases motility and generally physical activity, which leads to even higher degree of obesity. This vicious cycle is therefore self-perpetuating. Consequently, the first obvious negative consequence of energy surplus is surplus weight with its just described consequences. However, this is only one obvious consequence of energy surplus, which became a serious problem only recently. Besides overweight, an excess of visceral fat disturbs functioning of internal organs, also with deleterious consequences. Human beings like most of mammals evolved in environments in which availability of food underwent seasonal changes. Inhabitants of the Pacific Islands in particular were often forced to starve as a result of adverse winds. Recently, as food became widely available, they increasingly suffer from metabolic diseases, such as type II diabetes [50].

Equal dangers are observed even in slim individuals, which consume excessive amount of fats (including also cholesterol), built from predominantly saturated fatty acids. Some of them, however could show inherited problems with fat metabolism. The harmful phenomenon of the formation of atherosclerotic plaques is well known, but the awareness of the phenomena of lipotoxicity or glyco-lipotoxicity is much less widespread.

All extrinsic as well intrinsic factors increasing the level of diacylglycerides, ceramides or even fatty acyl CoA result in lipotoxicity, which is considered one of the main reasons o damage to kidney, pancreas and muscles [51]. An excess of saturated fatty acid-CoA inhibits transformation of toxic diacylgliceroles into three acyl glycerol molecules, which are stored in cells in the form of non-toxic fat droplets or in adipose tissue at the organismal level. Therefore, we have to clearly distinguish between dangers connected with obesity and overweight and the toxic effects of high amounts of consumed or endogenously generated saturated fatty acids. The ability to accumulate an excess of energy in the form of adipose tissue fats plays also an important positive role of the security valve. The disturbance in fatty acid metabolism, whether inherited or resulting from unbalanced feeding habits, can be, however, disastrous.

Such explanation of the effects of energy surplus and postulating its leading role in shortening life span can be considered too simple and trivial. It is obvious that some proximal causes of death cannot, at least directly, result from lipotoxicity and/or glyco/lipotoxicity or other effects of energy (lipid) excess, like atherosclerosis. However, their contribution to all cases of death is statistically enough significant to seriously consider such possibility. We simply evaluate the effects of any factor on longevity by studying proximal causes of death. It is well documented that diseases resulting from disturbed energy metabolism participate in most cases of death, even when they do not the direct cause of death. Such diseases, like cardiovascular, diabetes, renal, pancreatic and other disturbances, very often strongly contribute to the final effect. It is symptomatic that the studies on monkeys encompass measurements the body weight, glucose and fats levels and show serious overweight of fed *ad libitum* individuals. Centenarians, on the contrary, are without exception slim people.

## DATA SUPPORTING THE ENERGY SURPLUS HYPOTHESIS

### Behavior of NMRs in captivity strongly supports the energy surplus hypothesis

NMRs, when in captivity and supplied everyday with higher amount of food, live as long as in natural conditions and conserve late senescence phenotype. The only difference is that they are able to increase their rate of reproduction to reproduce every 80 days. Hence, these animals retained the ability of similar rodent species to assimilate higher amounts of food and their high rate of reproduction. Consequently, low frequency of reproduction in nature is evidently limited by food availability. The most important conclusion is that longevity of these animals is evidently encoded. It leaves a very low possibility of influencing it by feeding. Unfortunately, the data are not available for the effects of feeding according to *ad libitum* regime. Hence, the ability of investment of an excess of energy in reproduction can be treated as a security valve preventing negative effects of energy surplus. In contrast, other small rodent species already reproduce at the highest rate, and increased food supply could lead only to their accelerated death.

### Vegetative reproduction as an energy security valve in organisms which do not increase their size continuously

Lower animal organisms like cnidarians and others, besides sexual reproduction, reproduce also vegetatively by strobilation and budding, like for example Hydra. Their size remains comparatively constant, but an excess of energy can be directed to the formation of clones. Hence, an excess of energy is spent on vegetative reproduction instead of the body growth observed in lower vertebrates and crustaceans. Consequently, biological immortality of some of these organisms can result from some features like high degree of regenerative activity and effective energy dissipation, accompanied by simplicity of the body plan.

### The possibility that glyco/lipotoxicity plays a role in determining length of life is not restricted to mammals

This claim is supported by the fact that the deletion of genes coding for insulin-like regulatory pathways extends life span of even very dissimilar animals [52–55], such as invertebrates. In *Drosophila*, sugar and lipid metabolisms in the are regulated jointly. Although the exact mechanism extending the length of life is not fully understood, its existence strongly suggests that conserved mechanisms precisely regulating energy metabolism of the internal environment of multicellular organisms should exist. The diseases disturbing mechanisms of homeostasis of the internal environment strongly contribute to all causes of death [15]

## THE EFFECTS OF MEDITERRANEAN DIET

The effects of Mediterranean diet were considered as an argument supporting the free radical theory of aging because of high level of antioxidants characteristic of this diet. In addition, the presence of the monounsaturated fatty acids in olive oil suggests that those lipids that are resistant to oxidative damage molecules at the body temperature are safer than polyunsaturated fatty acids, which undergo lipid peroxidation process. Senescence marker, like lipofuscin accumulation, is a direct consequence of this destructive process [56, 57].

The omnipresence of lipofuscin deposits in postmitotic cells of numerous organisms needs explanation. But first, just like the imprecise term “aging” required disambiguation into senescence and longevity phenomena, the meaning of the term “senescence” also requires clarification. Accumulation of lipofuscins deposits concerns the cellular level. Long-lived NMRs or turtles are known to accumulate lipofuscins at the time when functional or macrostructural symptoms of aging are not visible [39]. Hence, delayed or negligible senescence of these organisms does not mean that the cellular level damage is not taking place. However, as long as the cells stay alive and function properly, the problem of this waste accumulation is less important. We propose to label these “cosmetic” symptoms as “cellular waste retention”. Hence, the presence of this very popular marker of senescence of mainly postmitotic cells does not seem to highly correlate with functional or macrostructural negative effects of the advanced age. They are rarely deleterious for the organism. The scale of free radical processes going on within the cell is, however, very low. The proof of it is that lipofuscin accumulation is diagnostic of postmitotic cells [57]. The dividing cells simply partition all waste products between the sibling cells (“dissolving” them).

The positive effects of high levels of monounsaturated fatty acids in olive oil, a dominating fat source of Mediterranean diet, can be however, explained by eliminating an excess of toxic diacylgliceroles, accompanied by lowering the level of saturated fatty acid CoA.

Lipotoxicity, which itself is presumably not connected with the free radical processes, destroys vital organs by a so far poorly understood mechanism. Life disturbing effects of senescence are instead manifested as extracellular deposits and concern such phenomena as atherosclerotic plaques and gall or renal stones.

The Mediterranean diet appears to have other rarely discussed effects. It is seldom noted that this diet is characterized by limited amount of calories and generally nutrients. The role of excess energy in contributing to the life shortening effects of our diet is also linked to supporting improper microbiome of our intestines. This factor is difficult to quantify, but it certainly influences quality of life.

Mass cell death is observed mainly in mesoderm [58]. It is executed at the cellular level, but its effects are manifested at the organismal level. These effects result mainly from intrinsic reasons, i.e., lack of telomerase, as suggested by the life-extending effects of its restoration at the organismal and tissue levels, not from free radical processes [59].

## POSSIBLE LIFE-EXTENDING STRATEGIES APPLICABLE TO HUMAN BEINGS

Therefore, the only strategy to survive till the age limit that is within an individual's control is a reasonable choice of lifestyle, mainly rational nutritional habits. However, medicine can also help in this case, either by lowering appetite, which has been additionally increased by taste enhancers widely present in processed food, or by helping in getting rid of various equally dangerous addictions. However, this approach will encounter opposition from powerful food processing concerns.

Medical interventions substantially increase our lifespan. Recent findings, however, strongly suggest that human aging is to a high extent dependent on epigenetic factors. One of the main age related diseases is type II diabetes, which either is the main reason of our death or strongly influences mortality from other diseases. For example, miR-194 or miR-143 strongly influences adipocyte differentiation [60–62]. Therefore, studies on the role of other micro RNA can be very important.

Recently, an entirely new approach to the aging problems appeared. The paper of Steve Horvath demonstrated that DNA methylation (DNAm) shows linear increase during human adulthood which perfectly correlates with the age of individuals [63]. Hence, besides postulated involvement of the TOR system [64, 65], epigenetic maintenance system (EMS), which additionally increases DNAm [63], also seems to be the area of factors responsible for senescence worth further exploration. The ticking rate of the epigenetic clock seems thus to be one of the main determinant of our aging. It would be possible to influence the activity of EMS at least in those regions of our genome which determine the most common causes of lowering of the most important human functions.

The ideas presented above clearly suggest that lowering the rate of senescence or extending life span within the inherited frame does not depend on a single process. Our life span is to a high extent inherited.

Consequently, in mammals aging can result from four independent factors:

- Not inhibited activity of various factors necessary during adolescence, symbolized by TOR and hyperfunction theory [6, 30].
- Glyco/lipotoxicity [51, 66]
- An excess of EMS system activity, eliminating or lowering expression of genes necessary for body maintenance of no longer growing adult organisms [67]
- Lowering the reproductive capacity of some somatic cells by lack of telomerase activity in some types of somatic cells because gene therapy appeared to increase life span of mice [68] and cultured human cells [69]
- Food excess might play some role in adversely affecting intestinal microbiome.

The hyperfunction theory postulates that during adulthood various biosynthetic processes lead to hypertrophy of somatic cells. NMRs however if better fed still conserve delayed senescence phenotype and high longevity. It suggests that the analysis of their organism reaction to availability of food can confirm the role of the TOR system in senescence or direct studies to new paths.

Sometimes, however, even the best correlation of various parameters can result from the action of another factor, not previously taken into account. To test for such a possibility, the studies on efficiency of EMS and DNAm in various long-lived organisms would be very informative. We therefore postulate to study both factors in organisms which show delayed senescence, like NMRs, in which neoplasia was eliminated. Equally informative could be studies on negligible senescence of those organisms which increase their body size throughout their whole life, like crocodilians and reptiles, which seem to be “young forever”. These organisms are comparatively closely related to humans. However, if the studies were to be extended to include completely different organisms which constantly grow, like crustaceans, the findings could be even more important. On the other extreme, the rate of DNAm in insects, which undergo complete metamorphosis from larvae through pupa till imago, can provide additional information and control. Soma of their imagoes is built exclusively from postmitotic cells and is in most part rebuilt during the pupa stage. Accumulation of these data will give an even better answer.

The studies on proximal causes of death of various long-lived organisms clearly show that only in one group of organisms, naked mole rats, neoplasia was eliminated as a cause of death [38]. In reptiles or long-lived fishes, living even longer than NMRs, tumors are encountered [70, 71]. It is possible that eliminating neoplasia became the most important aspect of the evolution of NMRs phenotype, because in other rodents neoplasia is rather common. Discovery of this so far unique characteristic of NMRs creates an opportunity for the study of the mechanisms preventing neoplasia [72].

## GLOSSARY OF THE USED TERMS

Aging: used scarcely in the paper, this term, describes all adverse for an organism effects accompanying the passing of time, such as increased probability of death (visualized by survival curves), cellular waste retention and senescence as organismal level effects, both structural and functional
Delayed senescence: late-onset organismal level symptoms of wear and tear
Free radicals: causative factors of damage, mainly reactive oxygen species ROS (not all of them are free radicals)
Longevity: a phenotype observed in species, populations and individuals which is characterized by longer than standard life, expressed in units of time. In case of species, longevity means extraordinarily long life, compared to other evolutionarily close species of similar size
Quasi-program: inherited trait which is a side effect of another genuine life program
Senescence: organismal level wear and tear processes manifested by mainly functional, anatomical and structural negative changes
Senescence markers: all symptoms indicating that an individual started aging. The term encompasses both organismal-level symptoms and cellular-level waste retention
Waste retention: accumulation within the cell of any unnecessary molecules or structures which are byproducts of various natural cellular processes (for example rDNA circles) or the products of damage like lipofuscins. These products can be either impossible to degrade or their accumulation is transient
Wear and tear: negative effects accompanying the passing of time concerning organismal level, both functional and anatomical/histological

## Abbreviations

DNAm: DNA methylation
EMS: epigenetic maintenance system
NMRs: naked mole rats
